# A Mechanism of Unidirectional Transformation, Leading to Antibiotic Resistance, Occurs within Nasopharyngeal Pneumococcal Biofilm Consortia

**DOI:** 10.1128/mBio.00561-18

**Published:** 2018-05-15

**Authors:** Santiago M. Lattar, Xueqing Wu, Jennifer Brophy, Fuminori Sakai, Keith P. Klugman, Jorge E. Vidal

**Affiliations:** a Hubert Department of Global Health, Rollins School of Public Health, Emory University, Atlanta, Georgia, USA; b Antibiotic Resistance Center, School of Medicine, Emory University, Atlanta, Georgia, USA; Louis Stokes Veterans Affairs Medical Center

**Keywords:** *Streptococcus pneumoniae*, antibiotic resistance, consortial biofilms, unidirectional transformation

## Abstract

Streptococcus pneumoniae acquires genes for resistance to antibiotics such as streptomycin (Str) or trimethoprim (Tmp) by recombination via transformation of DNA released by other pneumococci and closely related species. Using naturally transformable pneumococci, including strain D39 serotype 2 (S2) and TIGR4 (S4), we studied whether pneumococcal nasopharyngeal transformation was symmetrical, asymmetrical, or unidirectional. Incubation of S2^Tet^ and S4^Str^ in a bioreactor simulating the human nasopharynx led to the generation of Spn^Tet/Str^ recombinants. Double-resistant pneumococci emerged soon after 4 h postinoculation at a recombination frequency (rF) of 2.5 × 10^−4^ while peaking after 8 h at a rF of 1.1 × 10^−3^. Acquisition of antibiotic resistance genes by transformation was confirmed by treatment with DNase I. A high-throughput serotyping method demonstrated that all double-resistant pneumococci belonged to one serotype lineage (S2^Tet/Str^) and therefore that unidirectional transformation had occurred. Neither heterolysis nor availability of DNA for transformation was a factor for unidirectional transformation given that the density of each strain and extracellular DNA (eDNA) released from both strains were similar. Unidirectional transformation occurred regardless of the antibiotic-resistant gene carried by donors or acquired by recipients and regardless of whether competence-stimulating peptide-receptor cross talk was allowed. Moreover, unidirectional transformation occurred when two donor strains (e.g., S4^Str^ and S19F^Tmp^) were incubated together, leading to S19F^Str/Tmp^ but at a rF 3 orders of magnitude lower (4.9 × 10^−6^). We finally demonstrated that the mechanism leading to unidirectional transformation was due to inhibition of transformation of the donor by the recipient.

## IMPORTANCE

Pneumococcal transformation in the human nasopharynx may lead to the acquisition of antibiotic resistance genes or genes encoding new capsular variants. Antibiotics and vaccines are currently putting pressure on a number of strains, leading to an increase in antibiotic resistance and serotype replacement. These pneumococcal strains are also acquiring virulence traits from vaccine types via transformation. In this study, we recapitulated multiple-strain colonization with strains carrying a resistance marker and selected for those acquiring resistance to two or three antibiotics, such as would occur in the human nasopharynx. Strains acquiring dual and triple resistance originated from one progenitor, demonstrating that transformation was unidirectional. Unidirectional transformation was the result of inhibition of transformation of donor strains. Unidirectional transformation has implications for the understanding of acquisition patterns of resistance determinants or capsule-switching events.

## INTRODUCTION

Streptococcus pneumoniae (the pneumococcus) causes ~15 million cases of severe pneumococcal disease (PD) and nearly a half million deaths annually worldwide (1–5). Besides being a pathogen, the pneumococcus resides in the upper respiratory tract (i.e., oropharynx and nasopharynx) of most children under 5 years of age, without causing disease (6). While naturally residing in the human nasopharynx, pneumococcal resistance clones emerge through the acquisition of antibiotic resistance genes or through adaptation to antibiotic pressure (i.e., mutations) (7). Horizontal gene transfer (HGT) of antibiotic resistance genes occurs via mobile genetic elements (MGEs) or transformation. Mobile elements usually transfer genes conferring resistance to tetracycline (Tet), macrolides, including erythromycin (Ery), and/or efflux pumps, whereas recombination events via transformation lead to the acquisition of resistance mediated by mutations in the target site, such as resistance to β-lactams, streptomycin (Str), or trimethoprim (Tmp) (8, 9). Therefore, nasopharyngeal recombination via transformation has driven the recent spread of nonsusceptibility to β-lactam antibiotics, and resistance to trimethoprim (Tmp), within pneumococcal strains (10). The emergence of resistance of pneumococci to a new generation of antibiotics is expected to be driven by transformation. For example, mutations leading to resistance to linezolid and carbapenems have been recently described and may be spread by transformation (7, 11).

Genetic transformation was first observed by Griffith in 1928 while inoculating noncapsular, avirulent, pneumococci along with lysates from capsulated (i.e., virulent) colonies into mice, in order to recover—from dead mice—virulent capsule-expressing pneumococci (12). Recombination via transformation occurs through a genetically programmed and differentiated state called competence (13, 14). Competence can be induced *in vitro* (15) or “spontaneously” developed *in vivo* (12, 16). The mechanism is activated by a small peptide pheromone, called competence-stimulating peptide (CSP), which sequentially activates a cognate membrane receptor (ComD) and a response regulator (ComE). Genes encoding these proteins are located in an operon, including *comCDE*, where *comC* encodes CSP. S. pneumoniae strains produce different CSP pheromones, with the most common being CSP1 and CSP2. The membrane receptor, ComE, is specific for the CSP that the strain produces. In its natural environment, communication between pneumococci is restricted by the specificity of their CSPs, whereby cross talk only occurs between pneumococci secreting the same pherotype (13).

More than 100 genes are regulated via CSP during competence for transformation, including genes of the *comG* operon encoding type IV pilus (T4P) (17, 18). The T4P was recently demonstrated to be responsible for the uptake of naked DNA during transformation by strain R6, a D39 derivative, and TIGR4, although most genome-sequenced pneumococci carry the *comG* operon (19, 20). Within the *comG* operon, the first gene, *comGA*, encodes an ATPase required to produce pili, while the main pilin subunit is encoded by a downstream gene, *comGC*. A mutant lacking the ATPase or the main pilin subunit is unable to take up DNA by transformation (19).

While pneumococcal transformation occurs in the upper respiratory tract, it has traditionally been studied by incubating *in vitro*-generated competent pneumococci with purified DNA and synthetic CSP, perhaps because of the difficulties of recreating the nasopharyngeal microenvironment in the laboratory (13). Recently (2012), an *in vitro* model published by Marks et al. reproduced pneumococcal recombination between two transformable pneumococci, each carrying an antibiotic gene, and demonstrated that it occurred more efficiently in nasopharyngeal biofilms (21). The recombination frequency (rF) in this biofilm model ranged from 10^−3^ to 10^−4^ at 72 h postinoculation of human pharyngeal cells with two transformable pneumococcal strains (21).

Recent studies have demonstrated that children can be colonized by up to six pneumococcal strains at the same time, with ~50% of colonized children carrying at least two strains (22–24). With this high rate of multiple strain colonization, horizontal transference of genes among pneumococci is likely occurring frequently. It is therefore expected, although to the best of our knowledge not experimentally demonstrated, that homologous recombination of genes occurs symmetrically between naturally competent pneumococcal strains. In this study, we demonstrated that recombination between two transformable pneumococcal strains was unidirectional. The other strain, besides being competent for DNA uptake, acted as the donor.

## RESULTS

### Pneumococcal strains D39 (S2) and TIGR4 (S4) cohabit within biofilm consortia on human pharyngeal cells.

**FIG 1:**
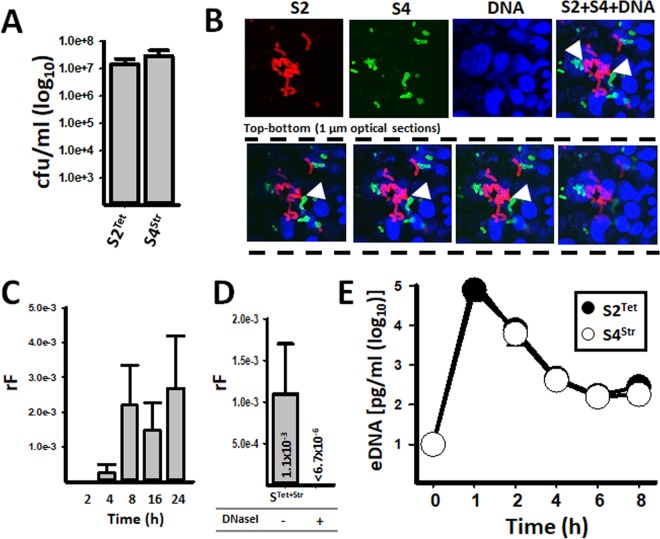
Rapid recombination of antibiotic resistance genes within pneumococcal biofilm consortia. Strains SPJV17 (S2^Tet^) and SPJV23 (S4^Str^) were inoculated into a bioreactor and incubated at ~35°C. After 8 h of incubation, (A) the density (CFU per milliliter) of each strain was obtained by culture in BAPs with the appropriate antibiotic, or (B) consortial biofilms were fixed with 2% PFA, stained with antiserotype-specific Alexa 555-labeled (S2) or Alexa 488-labeled (S4) antibodies, and the DNA was stained with DAPI. Preparations were analyzed by confocal microscopy. The top panels show xy optical middle sections of the indicated channel, or the merge, whereas bottom panels show representative xy 1-µm optical sections of a total of ~10 µm sectioned from top through bottom. (C) The recombination frequency (rF) of double-resistant pneumococci was obtained at each time point. (D) Bioreactor chambers were incubated for 8 h in the presence of 20 U/ml of DNase I (+) or left untreated (−), after which bacteria were counted and the rF was calculated. (E) Extracellular DNA (eDNA) was purified from supernatants collected from bioreactor chambers at the indicated time. The DNA was used as a template in serotype-specific qPCRs amplifying eDNA from either S2^Tet^ or S4^Str^. In panels A and C to E, error bars represent the standard errors of the means calculated using data from at least three independent experiments.

Recombination events leading to antibiotic resistance and capsule switching occur during pneumococcal nasopharyngeal carriage (22, 25, 26). We first investigated whether pneumococcal strains D39 (serotype 2 [for simplicity referred to as S2]) and TIGR4 (S4) can cocolonize in a nasopharyngeal biofilm consortium, and its ultrastructure was then imaged by confocal microscopy. To achieve this, we simulated a nasopharyngeal environment in a bioreactor where human pharyngeal cells were incubated for 8 h with a mixture of the two strains. After 8 h of incubation, the relative densities of S2 and S4 within the biofilm consortium were similar (Fig. 1A). To further visualize the localization of strains within the biofilm consortium, we conducted confocal studies, staining both strains with fluorescently labeled, serotype-specific, anti-S2 and anti-S4 antibodies, while the DNA was stained with DAPI (4′,6-diamidino-2-phenylindole). Figure 1B clearly shows S2 and S4 bacteria, both expressing their own capsule and forming aggregates of pneumococci, consistent with a biofilm consortium (Fig. 1B, top panels). Optical sections taken from the top of colonized pharyngeal cells, going down through the bottom, further revealed that the biofilm consortium was made of both strains integrated into a single structure with points of physical contact across the consortial biofilm (Fig. 1B, bottom panel). We hypothesize that the observed close proximity allows exchange of genetic material via transformation.

### Recombination of antibiotic resistance genes occurs early during the formation of biofilm consortia.

Given that antibiotics can be used to select for recombinant pneumococci, a time course study was conducted to investigate the timing of pneumococcal expression of resistance to two antibiotics within nasopharyngeal biofilm consortia. For these experiments, strains S2 and S4 were engineered to encode, in the chromosome, resistance to tetracycline (S2^Tet^), or streptomycin (S4^Str^). We also selected these strains because they produce different competence pheromones, CSP1 (27) or CSP2 (28), respectively, thus avoiding CSP-ComD (i.e., receptor) cross talk. Figure 1C shows that recombinant bacteria (Spn^Tet/Str^), i.e., resistant to both tetracycline and streptomycin, appeared soon after 4 h of incubation, reaching a maximum recombination frequency (rF) of 1.1 × 10^−3^ at 8 h postinoculation, after which the rF remained similar for up to 24 h (median rF, 2.0 × 10^−3^). Confirming that recombinant pneumococci emerged from transformation, Spn^Tet/Str^ bacteria were not obtained in bioreactor chambers incubated with DNase I (Fig. 1D). Moreover, double-resistant bacteria arose from recombination events, rather than from spontaneous mutations, since we did not obtain double-resistant Spn^Tet/Str^ pneumococci in bioreactor control chambers containing only S2^Tet^ or S4^Str^ (rF, <4.3 × 10^−7^ or <3.6 × 10^−8^, respectively). Sequencing confirmed the transference of streptomycin resistance-associated mutations (29) within the *rpsL* gene encoding ribosomal protein S12 in Spn^Tet/Str^ recombinants (not shown).

### Transformation leading to unidirectional acquisition of resistance occurs within pneumococcal biofilm consortia.

Both strains S2 (D39) and S4 (TIGR4) are transformable under standard transformation conditions, (27, 28). Accordingly, we obtained a similar transformation frequency (tF) when they were transformed with ~2.5 µg/ml of their own DNA (see Table S1 in the supplemental material) or each other’s DNA (i.e., S2 plus DNA from S4 [3.1 × 10^−7^] and S4 plus DNA from S2 [3.1 × 10^−6^]). We therefore hypothesized that recombinant Spn^Tet/Str^ bacteria would have arisen from both parents, whereby double-antibiotic-resistant pneumococci should belong to both serotype lineages S2^Tet/Str^ and S4^Tet/Str^. To test this hypothesis, 50 Spn^Tet/Str^ colonies were serotyped by conventional PCR (30) and Quellung reactions. All 50 recombinant bacteria, however, belonged to serotype 2 (i.e., S2^Tet/Str^). To screen for a larger number of recombinants, we designed a high-throughput assay utilizing serotype-specific quantitative PCRs (qPCRs). These reactions have a calculated limit of detection (LOD) of ~2 genome equivalents (22). To this end, we pooled all isolated colonies obtained in blood agar plates containing Tet and Str (~500 Spn^Tet/Str^ colonies from each plate), and DNA was extracted and utilized as the template in serotype-specific reactions. Using DNA template obtained from recombinant pneumococci, harvested from three independent experiments, serotype 2-specific reactions yielded a threshold cycle of detection (*C*_*T*_) value corresponding to ~7.8 × 10^9^ genome equivalents, whereas in serotype 4-specific reactions a *C*_*T*_ value was undetectable, confirming that recombinants were all S2^Tet/Str^ (see Table S2 in the supplemental material). S2^Tet/Str^ recombinants originated whether recombination took place on living cultures of human pharyngeal cells, immobilized human pharyngeal cells, or abiotic surfaces (Table S2). Altogether, this evidence suggested that unidirectional recombination occurred within pneumococcal biofilm consortia.

10.1128/mBio.00561-18.1 TABLE S1  Transformation frequency of competent pneumococci. Download TABLE S1, DOCX file, 0.1 MB. Copyright © 2018 Lattar et al. 2018 Lattar et al. This content is distributed under the terms of the Creative Commons Attribution 4.0 International license.

10.1128/mBio.00561-18.2 TABLE S2  Quantification of genome equivalents using serotype-specific qPCRs. Download TABLE S2, DOCX file, 0.1 MB. Copyright © 2018 Lattar et al. 2018 Lattar et al. This content is distributed under the terms of the Creative Commons Attribution 4.0 International license.

**FIG 2:**
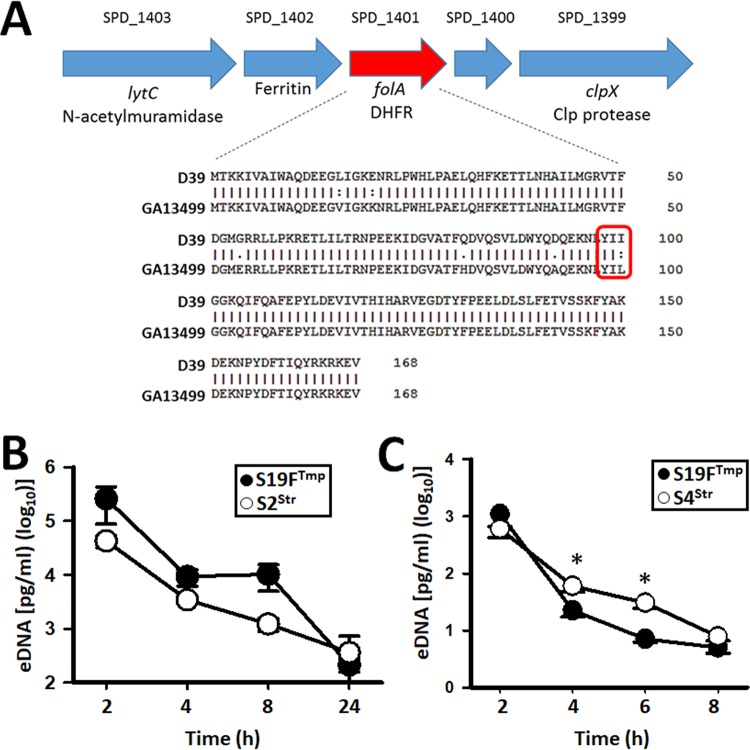
Unidirectional transformation occurs irrespective of CSP cross talk. (A) Genomic location of the *folA* gene in strain D39 (S2). The amino acid sequences of the (D39) trimethoprim-sensitive mature FolA protein and GA13499 (S19F) trimethoprim-resistant FolA protein are shown. Mutations associated with resistance to trimethoprim are indicated. Extracellular DNA (eDNA) was purified from supernatants collected from bioreactor chambers at the indicated incubation time. The eDNA was used as a template in serotype-specific qPCRs amplifying either (B) S2^Str^ or S19F^Tmp^ or (C) S4^Str^ or S19F^Tmp^. In panels B and C, error bars represent the standard errors of the means calculated using data from at least three independent experiments. *, statistical significance (*P* < 0.02).

The above results prompted us to test additional strains, including a genome-sequenced strain, GA13499 (serotype 19F), which, like D39, produces CSP1, thus allowing for cross talk between CSP1 pheromones and ComD receptors. GA13499 is naturally resistant to trimethoprim (Tmp^r^ [S19F^Tmp^]). Tmp resistance in S. pneumoniae has been associated with mutations within the *folA* gene, encoding dehydrofolate reductase, with a key mutation leading to an amino acid substitution at position 100: isoleucine to leucine (I→L) (31). Sequencing revealed that GA13499 contains mutations within *folA*, including the Tmp^r^-associated leucine substitution, whereas Tmp-susceptible (Tmp^s^) D39^Tet^ has an isoleucine (Fig. 2A). Strain S2^Tet^ was then incubated in the nasopharyngeal environment along with S19F^Tmp^ for 24 h, at which point Spn^Tet/Tmp^ recombinants were obtained at an rF of 1.5 × 10^−4^. Recombinants from three different experiments (~500 Spn^Tet/Tmp^ colonies from each) belonged to serotype 2 (i.e., S2^Tet/Tmp^), indicating that S2 strain acquired resistance to Tmp. We sequenced the *folA* gene in five of those S2^Tet/Tmp^ recombinant bacteria and confirmed that recombinants had acquired most mutations within the *folA* gene from S19F^Tmp^ (Fig. 2A).

Given that both S4^Str^ and S19F^Tmp^ acted as donors when incubated along with S2, we incubated in the bioreactor both donor strains and scored for resistance to Str and Tmp. Astonishingly, unidirectional transformation occurred leading to S19F^Tmp/Str^ again, although at a lower rF (4.9 × 10^−6^) compared to S2 derivative recipient strains.

**FIG 3:**
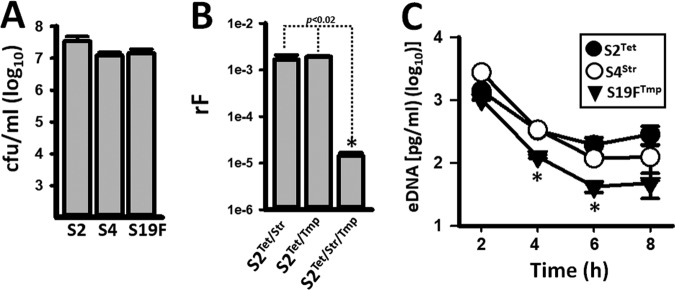
Unidirectional transformation occurs in biofilm consortia containing three strains. (A) Strains SPJV17 (S2), SPJV23 (S4), and GA13499 (S19F) were inoculated into a bioreactor and incubated at ~35°C for 8 h, after which both (A) the density (CFU per milliliter) of each strain was obtained by culture in BAPs with the appropriate antibiotic and (B) the recombination frequency (rF) of recombinants growing in BAPs with the indicated antibiotics was calculated. (C) Extracellular DNA (eDNA) was purified from supernatants collected from bioreactor chambers at the indicated incubation time and used as a template in serotype-specific qPCRs amplifying eDNA from either S2, S4, or S19F. Error bars represent the standard errors of the means calculated using data from at least three independent experiments. *, statistical significance (*P* < 0.05).

We then incubated the three strains together (i.e., S2^Tet^, S4^Str^, and S19F^Tmp^). The density of each strain was similar at 8 h postinoculation (Fig. 3A), and extracellular DNA (eDNA) from all three strains was detected in the supernatant (Fig. 3B). Recombinants belonged to S2. The rF of S2 that had acquired Str or Tmp resistance from S4^Str^ or S19F^Tmp^, respectively, was similar to that when only two strains were incubated together ~10^−3^ (Fig. 3B). The rF, however, significantly decreased (1.4 × 10^5^) when we scored for the acquisition of the two markers acquired from donors, Str and Tmp (Fig. 3B). Together these data confirmed a mechanism of unidirectional transformation within pneumococcal nasopharyngeal biofilm consortia leading to acquisition, via recombination, of antibiotic resistance.

### Both strains, the donor and the recipient, secrete eDNA into the supernatant.

A possible explanation for the unidirectional transformation observed within nasopharyngeal consortial biofilms could simply be the absence of spontaneous release of extracellular DNA (eDNA) by the recipient strain or increased release of eDNA by the donor. Therefore, we quantified eDNA, using serotype-specific qPCRs, in the supernatant of the bioreactor chambers inoculated with S2^Tet^ and S4^Str^. To minimize the presence of DNA at inoculation, inocula were prepared under conditions that were not permissive for competence, as described by Moscoso and Claverys (32). Before inoculation, pneumococci were washed three times with sterile culture medium. Even after these procedures, eDNA was still detectable in the supernatant of inocula from either S2^Tet^ or S4^Str^, although this residual DNA was detected at a very low concentration (~100 pg/ml [Fig. 1E]). Our experiments demonstrated, however, a marked increase in eDNA released from both strains after 1 h of incubation (Fig. 1E; see Table S3 in the supplemental material). At this time point, eDNA from S2^Tet^ (mean, 7.90 × 10^4^ pg/ml) was significantly higher (*P* = 0.023) than that from S4 (mean, 3.91 × 10^4^ pg/ml). This eDNA was able to transform competent pneumococci (either S2^Tet^ or S4^Str^); thereby it was permissive for transformation (not shown). The amounts of eDNA from both strains were then similar (*P* > 0.12) at 2, 4, 6 and 8 h postinoculation (Fig. 1E; Table S3). Overall, eDNA in the supernatant increased after 1 h postinoculation, and then its presence decreased—perhaps by degradation—with only 2.80 × 10^2^ pg/ml (median) from S2^Tet^ and 9.06 × 10^2^ pg/ml (median) from S4^Str^ detected in the supernatant at 8 h postinoculation (Fig. 1E; Table S3). Quantification of eDNA was also performed in supernatants of bioreactor chambers inoculated with S2^Tet^ and S19F^Tmp^, S4^Str^ and S19F^Tmp^, or S2^Tet^, S4^Str^, and S19F^Tmp^, with a similar peak of eDNA released by strains at 2 h postinoculation (Fig. 2B and C and Fig. 3C). This similar release of eDNA by strains in the biofilm consortium within 2 h of incubation, permissible for transformation (not shown), ruled out the possibility that unidirectional recombination was related to the availability of eDNA and indicated that spontaneous competence occurred.

10.1128/mBio.00561-18.3 TABLE S3  Quantification of eDNA in the supernatant of biofilm consortia made of S2^Tet^ and S4^Str^. Download TABLE S3, DOCX file, 0.1 MB. Copyright © 2018 Lattar et al. 2018 Lattar et al. This content is distributed under the terms of the Creative Commons Attribution 4.0 International license.

### Human pharyngeal cells trigger spontaneous competence within pneumococcal biofilm consortia.

Given that we did not add synthetic CSP to trigger competence, but recombination occurred within hours, we hypothesized that spontaneous competence occurred in the bioreactor. To investigate if pharyngeal cells, the culture medium, or the mammal serum used would trigger spontaneous competence, we inoculated strains S2^Tet^ and S4^Str^ into bioreactor chambers containing (i) living cultures of human pharyngeal cells, (ii) pharyngeal cells that had been immobilized with paraformaldehyde, and (iii) abiotic surfaces, all incubated with cell culture medium containing serum or (iv) living cultures of pharyngeal cells incubated with cell culture medium lacking serum. Our experiments demonstrated a similar rF when S2^Tet^ and S4^Str^ were incubated in the bioreactor with living cultures of pharyngeal cells (rF, 4.3 × 10^−4^), in pharyngeal cells that had been immobilized (rF, 1.2 × 10^−4^), and in cultures of pharyngeal cells incubated with medium without serum (rF, 6.2 × 10^−4^) (Table 1). In contrast, the rF was 3 orders of magnitude lower (rF, 3.3 × 10^−7^) when pneumococci were incubated on an abiotic surface (Table 1). Together these experiments demonstrated that spontaneous competence within pneumococcal biofilm consortia, leading to recombination of antibiotic resistance genes, is triggered upon contact between pneumococci and the host cell.

**TABLE 1:** Spontaneous competence occurs on human pharyngeal cells

Strains^a^	Substrate	Recombination frequency^b^
S2^Tet^ + S4^Str^	Pharyngeal cells	4.3 × 10^−4^ ± 2.1 × 10^−4^
S2^Tet^ + S4^Str^	Immobilized cells	1.2 × 10^−4^ ± 7.0 × 10^−5^
S2^Tet^ + S4^Str^	Abiotic	3.3 × 10^−7^ ± 4.1 × 10^−7^
S2^Tet^ + S4^Str^	Pharyngeal cells without serum	6.2 × 10^−4^ ± 3.8 × 10^−4^
S2^Tet^ + S19F^Tmp^	Pharyngeal cells	1.5 × 10^−4^ ± 9.2 × 10^−6^

a Str, streptomycin; Tet, tetracycline; Tmp, trimethoprim.

b Mean ± standard deviation from three independent experiments.

### Transformation of the donor is inhibited by the recipient strain by a mechanism different from Com.

**FIG 4:**
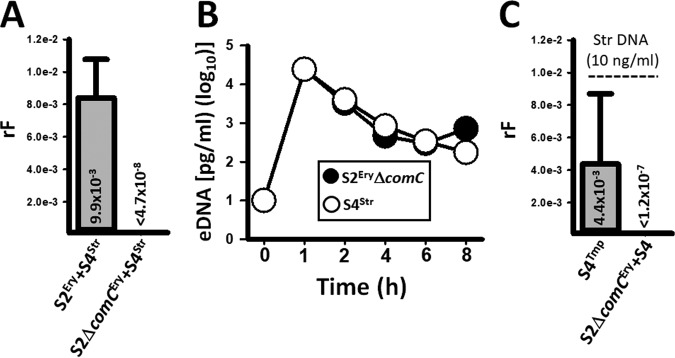
The recipient strain, S2, inhibits transformation of the S4 donor. (A) Strains D39^Ery^ (S2^Ery^) and TIGR4^Str^ (S4^Str^) or S2 Δ*comC*^Ery^ and S4^Str^ were incubated in the bioreactor for 8 h, after which (A) the recombination frequency (rF) was obtained. (B) Extracellular DNA (eDNA) was purified from supernatants collected from bioreactor chambers at the indicated time. The DNA was used as a template in serotype-specific qPCRs amplifying eDNA from either S2 Δ*comC*^Ery^ or S4^Str^. (C) Strain S4^Tmp^ or S4^Tmp^ and S2 Δ*comC*^Ery^ were inoculated into the bioreactor, which was perfused with cell culture medium containing 10 ng/ml of S2^Str^ DNA for 8 h. At the end of incubation, the rF was obtained. In all panels, error bars represent the standard errors of the means calculated using data from at least three independent experiments.

Strains S2, S4, and S19F are transformable *in vitro* (Table S1); however, in consortial biofilms only S2 acquired resistant determinants (19, 20). A possible explanation for the observed unidirectional transformation is that the recipient may have an increased transformation phenotype, or transformation of the donor is inhibited. If the first hypothesis is true, then removing CSP signaling in the recipient, and therefore competence for transformation, would now allow transformation of S4. To test this hypothesis, we incubated an S2^Ery^ Δ*comC* mutant, which is not transformable under standard transformation conditions, with S4^Str^ in the bioreactor. S2^Ery/Str^ recombinants were obtained when the S2^Ery^ wild type and S4^Str^ were incubated together for 8 h (Fig. 4A). In contrast, recombinants with resistance to both erythromycin and streptomycin were not harvested from bioreactor chambers incubated with S2^Ery^ Δ*comC* and S4^Str^ (Fig. 4A). This experiment revealed, as opposed to our original hypothesis, that reducing transformation of the recipient was not enough to allow transformation of a strain acting as a DNA donor.

Since we did not obtain recombinants, but we demonstrated that a similar amount of eDNA permissible for transformation from both strains was available during incubation in the bioreactor (Fig. 4B), we hypothesized that S2 inhibits transformation of S4. To investigate this hypothesis, we first asked whether S4 would become competent for DNA uptake in the simulated nasopharyngeal microenvironment by incubating strain S4^Tmp^ in the bioreactor along with 10 ng/ml of S2^Str^ DNA. Our experiments demonstrated a tF of 4.4 × 10^−3^ (Fig. 4C), which was 3 orders of magnitude different from the tF obtained in the conventional transformation assay done in a test tube, where synthetic CSP and pure DNA were added (Fig. 4C; Table S1). Therefore, S4^Tmp^ became naturally competent in the bioreactor. S4 was then incubated in the presence of S2^Str^ DNA (10 ng/ml), but this time we also inoculated the nontransformable S2^Ery^ Δ*comC* strain. Surprisingly, S4 transformants were not obtained, indicating that the recipient, S2, inhibited transformation of the donor. Several attempts were made to conclude that the supernatant did not inhibit transformation of the donor (not shown). Therefore, the potential mechanism appears to be independent of CSP production and contact mediated.

## DISCUSSION

In this study, we recreated nasopharyngeal recombination of antibiotic resistance genes between pneumococcal strains and we demonstrated that it occurs very rapidly—within 4 h postcontact of pneumococci with human pharyngeal cells. Our experiments also found, as revealed by antibiotic selection, that unidirectional transformation occurred within pneumococcal biofilm consortia, leading to the rise of a single lineage of highly transformable pneumococci. Antibiotic selection was used as a surrogate for the pressure that, in the pneumococcal vaccine age, is challenging pneumococcal strains.

Evidence presented in this study, and elsewhere (26), indicates that pherotype cross talk is not involved in unidirectional transformation. For example, we demonstrated here that regardless of whether CSP-ComD receptor cross talk was allowed, unidirectional transformation occurred. This evidence correlated with the absence of transformation of the donor even when it was incubated with a CPS knockout recipient. Moreover, observations from whole-genome sequence studies conducted on prevaccine pneumococcal isolates demonstrated that pherotypes CSP1 and CSP2 or their variants do not account for recombination differences among strains, suggesting that the mechanism leading to unidirectional transformation occurs irrespective of CSP cross talk (26).

Genomic studies have demonstrated that resistance to antibiotics among pneumococcal strains is driven by expansion of clones that have acquired resistance phenotypes by HGT, more than by *de novo* mutations (10). The mechanism is very efficient given the high resistance rates, and except for transposon-mediated resistance that will be discussed below, it includes resistance to β-lactams, trimethoprim, and some of the last resort antibiotics, such as linezolid and carbapenems (7). The DNA taken by transformation is in general small since it appears to be cleaved into ~6.6-kb fragments (33). These fragments undergo homologous recombination to integrate, into the genome, DNA pieces of ~4.4 kb, calculated using data from species-wide multilocus sequence typing studies (34), or ~6.3 kb, as demonstrated by sequencing (35). A more recent study demonstrated heterogeneity in pneumococcal recombination with microrecombination fragments of ~0.03 to 0.6 kb and macrorecombination fragments ranging from 9 to 10 kb (36). Secondary, nonselective, recombination events via unidirectional transformation were not investigated in our study but have been calculated by Croucher et al. to be ~2.3 kb (37).

It has been well documented that mobile genetic elements (MGEs [e.g., transposons]) carry genes for resistance to tetracyclines and macrolides. These MGEs are usually ~20 kb or larger and are therefore not simply transferred by transformation (38). More recently, capsular switch events were linked to mobilization of the whole capsule locus from a nonvaccine type donor, 35B/ST558, to a vaccine type recipient, 9V/ST156, leading to a new lineage of capsule switch variants belonging to serotype 35B/ST156 (39). Evidence of *in vitro* transference of Tn-encoded resistance, or capsule genes, in pneumococcal strains is not available yet, but such transference is currently being investigated in our laboratories.

Another important contribution in this article refers to the development of spontaneous competence. Our experiments demonstrated a burst of eDNA released into the supernatant as soon as 1 h postinoculation of human pharyngeal cells, in all different mixtures of pneumococcal strains tested. Release of eDNA has been linked to the development of competence, in studies conducted by Moscoso and Claverys (32), and also to production of bacteriocins and other fratricide factors, whose secretion causes heterolysis and therefore the release of DNA (40–42). Release of abundant eDNA in the bioreactor was also observed when strains were inoculated alone (data not shown); thereby, it is unlikely that such amounts of DNA were generated by heterolysis but rather by a mechanism coupled to spontaneous competence. Release of DNA early during colonization may also help pneumococci to attach to host cells and/or form bacterial aggregates leading to nasopharyngeal biofilms.

We, and others, have clearly demonstrated that under static culture conditions irreversible autolysis occurs in monostrain pneumococcal biofilms (40, 43). Heterolysis also happened in consortial biofilms inoculated with two different strains incubated under static conditions (42, 44). All of this evidence correlates with the finding that release of DNA in the supernatants is highest in stationary cultures because it comes from lysis of pneumococci (32). This, however, did not occur in our bioreactor model, where the sharpest peak of eDNA in the supernatants was detected during the log phase, within 2 h postinoculation. Studies by Wholey et al. demonstrated that heterolysis in consortial biofilms is linked to production of the BlpC bacteriocin, whose synthesis and release are controlled by the competence system (42). In the above-mentioned study, inoculation of strains at a 1:1 ratio was enough to completely eradicate susceptible pneumococci by a BlpC-producing strain (42). A decrease in density, but by no means eradication of a pneumococcal strain, was observed in recent studies in which two strains were inoculated at a similar density and cultured under static conditions (44). Moreover, when inoculated at similar densities in the bioreactor the density of strains was similar throughout the incubation period. Therefore, unidirectional transformation leading to a rapid acquisition of genes by recombination does not appear to be simply the eradication of one of the strains in the consortial biofilms. We cannot rule out the possibility that killing of a fraction of the population of the recipient or the donor took place before the burst of released eDNA and the appearance of recombinants.

Remarkably, rapid development of spontaneous competence caused nearly 1 in 1,000 pneumococci to acquire antibiotic resistance within 4 h. High rF (e.g., ~10^−3^) was consistently obtained in experiments where the resistance determinant included specific mutations (i.e., *folA* and *rpsL*), whereas the transference of whole resistance genes, such as *tetM* or *ermB* was observed at a lower rF (usually >10^−4^). These observations were similar to those described in a mouse model of colonization (45). In this mouse model, recombinants were harvested 48 h postinoculation at an rF similar to that obtained in the bioreactor (45). Together this evidence suggests that acquisition of DNA among pneumococci and the development of competence occur soon after pneumococci colonize the host.

What stimulated the rapid development of competence? To the best of our knowledge, the only molecule from the host that has been identified as a trigger for recombination is chitin, an oligosaccharide found in the exoskeletons of crustaceans, the natural habitat of Vibrio cholerae strains (46). Chitin induced an upregulated production of the V. cholerae T4P (47), used for DNA uptake, and proteins of the transformation machinery (48). More recent studies showed that chitin induces production of a type 6 secretion system (T6SS), which is utilized by V. cholerae to kill its neighbors and thus allow release of DNA, which is then taken by the transformation machinery, for recombination (49). While pneumococcus utilizes a T4P to take up DNA, a T6SS has not yet been reported, and a source for chitin in the bioreactor, the mouse model, or the human host, is unlikely.

Attempts were made in this study to begin to understand the development of such early “spontaneous” competence leading to nasopharyngeal recombination. The outcome of our extensive experimentation was that recombination of antibiotic resistance determinants, whose mechanism included release of eDNA and uptake (e.g., spontaneous competence), only required contact with host cells. Experiments by Marks et al. also demonstrated recombination in a biofilm model utilizing paraformaldehyde (PFA)-fixed pharyngeal cells in comparison to abiotic surfaces (45). We ruled out the possibility that a secreted product from human cells ignited pneumococcal recombination, given that the rF obtained in experiments using immobilized cells was similar to that obtained in living cultures of pharyngeal cells. Neither the cell culture medium nor the mammal serum utilized had an effect on the rapid pneumococcal recombination observed. As such, pneumococcal strains inoculated in the bloodstream of mice were not able to acquire DNA that was concomitantly inoculated in the seminal experiments published by Griffith in 1928 (12). Likewise, a high recombination frequency was not observed when two pneumococcal strains were inoculated in a mouse model of sepsis in more modern studies of pneumococcal recombination by the group of A. Hakansson (21).

So far, we have not been able to obtain recombinants within 8 h of incubation in a static plate model, whether or not it contains human pharyngeal cells (40, 43). The absence of recombination may be because of the heterolysis phenotype reported using static incubation conditions (42, 44) or the accumulation of DNases in static cultures, which may degrade available eDNA for transformation (32). In summary, we have demonstrated in this study that unidirectional transformation occurred within pneumococcal biofilm consortia and that unidirectional transformation is mediated by inhibition of transformation within pneumococcal nasopharyngeal biofilms.

## MATERIALS AND METHODS

### Bacterial strains, culture media, and antibiotics.

The S. pneumoniae strains used in the present study are listed in Table 2. Strains were routinely cultured on blood agar plates (BAPs) or grown in Todd-Hewitt broth containing 0.5% (wt/vol) yeast extract (THY) at 37°C with a 5% CO_2_ atmosphere. Where indicated, streptomycin (Str; 200 µg/ml), trimethoprim (Tmp; 10 µg/ml), tetracycline (Tet; 1 µg/ml), and/or erythromycin (Ery; 1 µg/ml) was added to the BAP. All antibiotics were purchased from Millipore-Sigma (Saint Louis, MO).

**TABLE 2:** Strains used in this study

S. pneumoniae strain	Description, relevant genotype, or phenotype^a^	Reference or source
D39	Avery strain, serotype 2, CSP1	56
SPJV01	D39 carrying pMV158GFP, Tet^r^	51
SPJV10	D39 Δ*comC*	43
SPJV17	D39 carrying *tetM* gene in chromosome, Tet^r^	This study
SPJV22	D39 Str^r^	This study
TIGR4	Invasive isolate, serotype 4, CSP2	28
SPJV23	TIGR4 Str^r^	This study
SPJV27	TIGR4 Tmp^r^	This study
SPJV28	TIGR4 carrying *tetM* gene in chromosome, Tet^r^	This study
SPJV29	D39 Tmp^r^	This study
GA13499	Serotype 19F, Tmp^r^ CSP1	57

a Str^r^, streptomycin resistant; Tet^r^, tetracycline resistant; Tmp^r^, trimethoprim resistant.

### Preparation of the inoculum to produce pneumococcal biofilm consortia.

The inoculum was prepared as previously described (43). Briefly, an overnight BAP culture was used to prepare a cell suspension in THY broth to an optical density at 600 nm (OD_600_) of 0.05. This suspension was incubated at 37°C in a 5% CO_2_ atmosphere until the culture reached an OD_600_ of ~0.2 (early log phase), and then glycerol was added to a final concentration of 10% (vol/vol) and stored at −80°C until used. A frozen aliquot from each batch was removed to obtain the density (CFU per milliliter) by dilution and plating.

### Preparation of antibiotic-resistant, D39 derivative, and TIGR4 derivative pneumococcal strains.

SPJV17 and SPJV28 were constructed by transforming D39 or TIGR4, respectively, with integrative plasmid pPP2, which targeted *tetM* to the nonessential *bgaA* gene (50). SPJV22 and SPJV23 were transformed with DNA from strain R6Ami9 encoding resistance to streptomycin (44). Strains SPJV27 and SPJV29 were prepared by transformation of TIGR4 or D39, respectively, with DNA from GA13499 encoding resistance to trimethoprim. Chromosomal integration of the *tetM* gene was confirmed by PCR with primers JVS101L and JVS102R. Mutations within *folA* or *rpsL*, conferring resistance to Tmp or Str, respectively, were confirmed by sequencing with primer JVS99L or JVS100R for Tmp and with primer JVS103L or JVS104R for Str. Transformation was done following standard methods (51, 52).

### Cell cultures.

Human pharyngeal Detroit 562 cells (ATCC CCL-138) were cultured in Eagle’s minimum essential medium (EMEM; Lonza, Walkersville, MD) supplemented with non-heat-inactivated 10% fetal bovine serum (FBS; Atlanta Biologicals, Flowery Branch, GA), 1% nonessential amino acids (Millipore-Sigma, Saint Louis, MO), 1% glutamine (Millipore-Sigma, Saint Louis, MO), penicillin (100 U/ml), and streptomycin (100 µg/ml), and the pH was buffered with HEPES (10 mM; Gibco, Thermo Fisher Scientific, Grand Island, NY). Cells were harvested with 0.25% trypsin (Gibco, Thermo Fisher Scientific, Grand Island, NY), resuspended in the cell culture medium, and incubated at 37°C in a 5% CO_2_ humidified atmosphere.

### Inoculation of the bioreactor with pneumococcal strains.

Detroit 562 cells (ATCC CCL-138) were grown on Snapwell filters (Corning, Corning, NY); these filters have a polyester membrane (0.4 µm) supported by a detachable ring. Once polarized (4 to 5 days), Snapwell filters containing pharyngeal cells were immediately placed in a sterile vertical diffusion chamber (bioreactor) (43). Where specified, a set of pharyngeal cells were prefixed with 2% paraformaldehyde (Millipore-Sigma, Saint Louis, MO) for 15 min, followed by extensive washing with sterile phosphate-buffered saline (PBS), prior to installation in the bioreactor chambers. To create an abiotic surface, some bioreactor chambers were installed with Thermanox coverslips (Thermo Fisher Scientific, Grand Island, NY). The bioreactor chamber has an inlet from which the apical side (inner chamber) was perfused at a low flow rate of ~0.20 ml/min with sterile EMEM, which contained 5% FBS but no antibiotics, using a Master Flex L/S precision pump system (Cole-Parmer, Vernon Hills, IL). Where indicated, DNase I was added to a final concentration of 50 U/ml. Perfused culture medium and planktonic cells exit the bioreactor chamber by a parallel outlet located on top of the chamber.

Bioreactor chambers were then inoculated with ~1 × 10^6^ CFU/ml of each pneumococcal strain and incubated at ~35°C under a sterile environment. At the end of the incubation period, Snapwell inserts or Thermanox was removed, and biofilm consortia were analyzed as follows. Biofilm consortia or monostrain biofilms (control) were harvested by sonication for 15 s in a Bransonic ultrasonic water bath (Branson, Danbury, CT), followed by extensive pipetting to remove all attached bacteria. An aliquot was used to obtain the density of each strain, by dilution and plating in BAP containing the appropriate antibiotic, and another aliquot was directly plated onto BAP containing two or three antibiotics to recover recombinant pneumococci.

### Calculation of rF and tF.

The density of parent strains was counted in BAPs containing one antibiotic, while recombinants were counted on BAPs with two or three antibiotics. The recombination frequency (rF) was the density of pneumococci with dual or triple markers divided by the density of the parent strain. The transformation frequency (tF) was the number of transformants relative to the total pneumococci recovered in the transformation reaction.

### Confocal micrographs of pneumococcal biofilm consortia.

To visualize biofilm consortia by confocal microscopy, we installed a glass coverslip inside the Snapwell filters prior to seeding with human pharyngeal cells. Once pharyngeal cells became polarized, the Snapwell filter was installed in the bioreactor and inoculated as described above. At the end of the incubation, the coverslips containing pharyngeal cells with pneumococcal consortial biofilms were washed twice with PBS and fixed with 2% PFA for 15 min at room temperature. Once the fixative agent was removed, cells were washed with PBS and blocked with 2% bovine serum albumin (BSA) for 1 h at room temperature. These cells containing consortial biofilms were then incubated with serotype-specific polyclonal antibodies (~40 µg/ml; Statens Serum Institute, Copenhagen, Denmark) for 1 h at room temperature. Antibodies had been previously labeled with Alexa 488 (anti-S4) or Alexa 555 (anti-S2) following the manufacturer’s recommendations (Molecular Probes, Thermo Fisher Scientific, Grand Island, NY) (44). Stained preparations were finally washed two times with PBS and were mounted with ProLong Diamond antifade mountant with DAPI (Molecular Probes, Thermo Fisher Scientific, Grand Island, NY). Confocal images were obtained using an Olympus FV1000 confocal microscope and were analyzed with ImageJ version 1.49k (National Institutes of Health) or Imaris software (Bitplane, South Windsor, CT).

### High-throughput assay for pneumococcal serotyping.

Recombinant pneumococci obtained in BAPs containing two or three antibiotics were pooled in 200 µl of sterile PBS, and DNA from this population was purified as detailed below. This DNA was utilized as the template for serotype-specific quantitative PCRs with primers and probes listed in Table 3. Reactions targeted serotype-specific sequences within the capsule polysaccharide (*cps*) locus of each serotype (22, 53) and were run along serially diluted DNA standards corresponding to 4.29 × 10^5^, 4.29 × 10^4^, 4.29 × 10^3^, 4.29 × 10^2^, 4.29 × 10^1^, and 2.14 × 10^1^ genome equivalents per reaction (54). Reactions were carried out using a Bio-Rad CFX96 Touch real-time PCR detection system (Bio-Rad, Hercules, CA) with the following cycling parameters: 50°C for 2 min, 95°C for 2 min, and 40 cycles of 95°C for 15 s and 60°C for 1 min. The standard curve and regression equation obtained were then used to calculate final genome equivalents per milliliter using the CFX software (Bio-Rad, Hercules, CA).

**TABLE 3:** Primers and qPCR assays used in this study

Primer or assay	Sequence (5′→3′)	Reference(s)
Primers		
2 f	TATCCCAGTTCAATATTTCTCCACTACACC	30
2 r	ACACAAAATATAGGCAGAGAGAGACTACT	
4 f	CTGTTACTTGTTCTGGACTCTCGATAATTGG	30
4 r	GCCCACTCCTGTTAAAATCCTACCCGCATTG	
JVS99L	TTGCCAGCAGAATTGCAGCA	This study
JVS100R	AAATAGGTATCTCCTTCCACC	This study
JVS101L	CTGCTGGGGTACTAACAGGG	This study
JVS102R	CGGCACTTCGATGTGAATGG	This study
JVS103L	ATCTTGACAAGCAAGGGAAAAT	This study
JVS104R	TTCCTTATGCTTTTGGACGTTT	This study
PCR assays		
Serotype 2 Fwd	TTATGGACTGGCTGATGGTTCTC	22, 58
Serotype 2 Rev	AAATCCTGACCCAATAATAGCCTTT	
Serotype 2 probe^a^	AGGTCAACGTATTGGAACTCTTAGAAATTGGGAAA	
Serotype 4 Fwd	TGGGATGACATTTCTACGCACTA	22, 58
Serotype 4 Rev	CCGTCGCTGATGCTTTATCA	
Serotype 4 probe^a^	TCCTATTGGATGGTTAGTTGGTGA	
Serotype 19F Fwd	GGTCATGCGAGATACGACAGAA	22, 58
Serotype 19F Rev	TCCTCATCAGTCCCAACCAATT	
Serotype 19F probe^a^	ACCTGAAGGAGTAGCTGCTGGAACGTTG	

a Probes are labeled 5′ with 6-carboxyfluorescein (FAM) and 3′ with black hole quencher 1 (BHQ1).

### DNA extraction.

DNA was extracted from 200 µl of a fresh suspension of pneumococcal strains with the QIAamp DNA minikit (Qiagen, Valencia CA) according to the manufacturer’s instructions. Final elution was done with 100 µl of elution buffer. DNA preps were quantified using a NanoDrop spectrophotometer and stored at −80°C until used.

### Quantification of eDNA.

Supernatants were collected from the outlet of bioreactor chambers, centrifuged for 15 min at 14,000 × *g* in a refrigerated centrifuge (Eppendorf, Hauppauge, NY), and then sterilized with a 0.45-µm-pore syringe filter. This bacterium-free supernatant was DNA extracted using the QIAamp DNA minikit following the manufacturer’s instructions. Purified DNA was used as the template in serotype-specific quantitative PCRs (qPCRs) using the primer and probe sets shown in Table 3. Reactions were performed essentially as described as above and in our previous studies (22, 55). For eDNA quantification purposes, standards containing 1 × 10^3^, 1 × 10^2^, 1 × 10^1^, 1 × 10°, 1 × 10^−1^, 5 × 10^−2^, or 1 × 10^−3^ pg of chromosomal DNA from the appropriate serotype were run in parallel to generate a standard curve. This standard curve was then used to calculate the eDNA concentration using the Bio-Rad CFX Manager software.

### Serotype-specific conventional PCRs.

Serotype-specific PCRs were performed in 25-µl volumes containing ~100 ng DNA or 3 µl of bacterial lysate, 1 µM serotype-specific forward or reverser primer listed in Table 3, and 1× the PCR master mix from the Qiagen Multiplex PCR kit (Qiagen, Valencia CA). Reactions were run using the following cycling parameters: 1 cycle at 95°C for 15 min, followed by 35 cycles of 94°C for 30 s, 54°C for 1 min, and 72°C for 1 min, with a final extension of 72°C for 10 min. Products were run on 2% agarose gels, stained with SYBR Safe DNA gel stain (life technologies, Grand Island, NY), and visualized under a UV transilluminator (Bio-Rad, Hercules, CA).

### Transformation reactions.

S. pneumoniae strains were made competent using standard procedures and then transformed with 500 ng of pure DNA containing 100 ng of competence-stimulating peptide 1 (CSP1 [EMRLSKFFRDFILQRKK]) or CSP2 (EMRISRIILDFLFLRKK) in a reaction volume of 200 µl (15). CSP1 and CSP2 were synthesized at Millipore-Sigma (Saint Louis, MO).

### Sequencing reactions.

Purified DNA from wild-type strains or recombinant derivatives was used as the template to PCR amplify the *folA* gene using primers JVS99L and JVS100R listed in Table 3. PCR products were purified using the QIAquick PCR purification kit (Qiagen, Valencia, CA). Both DNA strands (5′→3′ or 3′→5′) were sequenced, in separate reactions, at Eurofins Genomics (Eurofins, Louisville, KY). Sequences were analyzed using Lasergene 10 version 10.1.1(3) (DNASTAR, Madison, WI).

### Statistical analysis.

Statistical analysis presented in this study was performed using the Mann-Whitney *U* test and the software SigmaPlot version 12.0 (Systat Software, Inc., San Jose, CA).
