# Supplementary material for: A Mechanism of Unidirectional Transformation, Leading to Antibiotic Resistance, Occurs within Nasopharyngeal Pneumococcal Biofilm Consortia
Source: mBio. 2018 May 15;9(3):e00561-18. doi: 10.1128/mBio.00561-18 (PMC5954218; doi:10.1128/mBio.00561-18)
Supplement: TABLE S1 [file mbo003183889st1.docx]

| **Strain** | **DNA (~2.5 µg/ml) from** | **Transformation frequency** |
| --- | --- | --- |
| S2^Tet^ | S2^Str^ | 5.3x10^-6^±2.4x10^-6^ |
|  | S4^Str^ | 9.8x10^-6^±7.5x10^-6^ |
|  | S4^Ery^ | 3.1x10^-7^±4.6x10^-8^ |
|  | S19F^Tmp^ | 4.8x10^-6^±1.5x10^-6^ |
| S4^Tet^ | S4^Str^ | 1.9x10^-6^±1.7x10^-6^ |
| S4^Str^ | S2^Tet^ | 3.1x10^-6^±1.2x10^-6^ |
|  | S2^Ery^ | 3.3x10^-6^±2.2x10^-6^ |
|  | S19F^Tmp^ | 2.2x10^-5^±6.3x10^-6^ |
| S19F^Tmp^ | S2^Tet^ | 5.48x10^-7^±1.1x10^-8^ |

**Supplemental Table 1. Transformation frequency of competent pneumococci.**

±Standard deviation of three independent experiments. Streptomycin (Str), erythromycin (Ery), tetracycline (Tet).
