# Supplementary material for: A Mechanism of Unidirectional Transformation, Leading to Antibiotic Resistance, Occurs within Nasopharyngeal Pneumococcal Biofilm Consortia
Source: mBio. 2018 May 15;9(3):e00561-18. doi: 10.1128/mBio.00561-18 (PMC5954218; doi:10.1128/mBio.00561-18)
Supplement: TABLE S2 [file mbo003183889st2.docx]

**Supplemental Table 2. Quantification of genome equivalent using serotype-specific qPCR reactions**

| **Strains** | **Spn^Tet/Str^ obtained from bioreactor with:** | **Serotype (S)-specific reaction**  **(genome equivalent/ml)** | |
| --- | --- | --- | --- |
|  |  | **S2** | **S4** |
| S2^Tet^+S4^Str^ | Pharyngeal cells | 7.8x10^9^±5.7x10^8^ | <400* |
| S2^Tet^+S4^Str^ | Immobilized pharyngeal cells | 2.7x10^9^±1.6x10^9^ | <400 |
| S2^Tet^+S4^Str^ | Polystyrene | 8.5x10^9^±1.0x10^10^ | <400 |
| S2^Tet^+S4^Ery^ | Pharyngeal cells | 2.5x10^9^±5.6x10^9^ | <400 |
|  |  |  |  |
|  |  | **S2** | **S19F** |
| S2^Tet^+S19F^Tmp^ | Pharyngeal cells | 1.6x10^9^±2.1x10^9^ | <400* |
|  |  | **S4** | **S19F** |
| S4^Str^+S19F^Tmp^ | Pharyngeal cells | <400 | 1.02x10^8^±9.8x10^7^ |

*Limit of detection of S4-specific, or S19F-specific, qPCR reaction. ± Standard deviation obtained from three independent experiments.
