# Supplementary material for: A Mechanism of Unidirectional Transformation, Leading to Antibiotic Resistance, Occurs within Nasopharyngeal Pneumococcal Biofilm Consortia
Source: mBio. 2018 May 15;9(3):e00561-18. doi: 10.1128/mBio.00561-18 (PMC5954218; doi:10.1128/mBio.00561-18)
Supplement: TABLE S3 [file mbo003183889st3.docx]

**Supplemental Table 3. Quantification of eDNA in the supernatant of biofilm consortia made of S2^Tet^ and S4^Str^.**

| **S2^Tet^ +S4^Str^** | **eDNA (pg/ml)*** | ***p***** |
| --- | --- | --- |
| S2^Tet^ (Time, h) |  |  |
| 1 | 7.90x10^4^±3.3x10^3^ | 0.023 |
| 2 | 7.77x10^3^±3.8x10^3^ | 0.413 |
| 4 | 4.71x10^2^±1.4x10^2^ | 0.207 |
| 6 | 1.73x10^2^±3.7x10^1^ | 0.122 |
| 8 | 2.86x10^2^±1.2x10^2^ | 0.152 |
| S4^Str^ (Time, h) |  |  |
| 1 | 3.91x10^4^±1.9x10^4^ |  |
| 2 | 5.13x10^3^±3.3x10^3^ |  |
| 4 | 9.77x10^2^±5.7x10^2^ |  |
| 6 | 6.98x10^2^±4.6x10^2^ |  |
| 8 | 9.06x10^2^±5.9x10^2^ |  |

*mean±standard deviation

**Student *t* test, in comparison with the same time point of S4^Str^
